# A multiplex assay for the simultaneous detection of antibodies against 15 *Plasmodium falciparum *and *Anopheles gambiae *saliva antigens

**DOI:** 10.1186/1475-2875-9-317

**Published:** 2010-11-08

**Authors:** Elena Ambrosino, Chloé Dumoulin, Eve Orlandi-Pradines, Franck Remoue, Aissatou Toure-Baldé, Adama Tall, Jean Biram Sarr, Anne Poinsignon, Cheikh Sokhna, Karine Puget, Jean-François Trape, Aurélie Pascual, Pierre Druilhe, Thierry Fusai, Christophe Rogier

**Affiliations:** 1 IRBA & UMR6236, Marseille, France; 2 Institut Pasteur, Paris, France; 3 IRD, Montpellier, France; 4 Institut Pasteur de Dakar, Dakar, Senegal; 5 IRD, Dakar, Senegal and UMR6236, Marseille, France; 6 ONG Espoir pour la Santé, St-Louis du Sénégal; 7 GENEPEP, Montpellier France

## Abstract

**Background:**

Assessment exposure and immunity to malaria is an important step in the fight against the disease. Increased malaria infection in non-immune travellers under anti-malarial chemoprophylaxis, as well as the implementation of malaria elimination programmes in endemic countries, raises new issues that pertain to these processes. Notably, monitoring malaria immunity has become more difficult in individuals showing low antibody (Ab) responses or taking medications against the *Plasmodium falciparum *blood stages. Commonly available techniques in malaria seroepidemiology have limited sensitivity, both against pre-erythrocytic, as against blood stages of the parasite. Thus, the aim of this study was to develop a sensitive tool to assess the exposure to malaria or to bites from the vector *Anopheles gambiae*, despite anti-malarial prophylactic treatment.

**Methods:**

Ab responses to 13 pre-erythrocytic *P. falciparum*-specific peptides derived from the proteins Lsa1, Lsa3, Glurp, Salsa, Trap, Starp, CSP and Pf11.1, and to 2 peptides specific for the *Anopheles gambiae *saliva protein gSG6 were tested. In this study, 253 individuals from three Senegalese areas with different transmission intensities and 124 European travellers exposed to malaria during a short period of time were included.

**Results:**

The multiplex assay was optimized for most but not all of the antigens. It was rapid, reproducible and required a small volume of serum. Proportions of Ab-positive individuals, Ab levels and the mean number of antigens (Ags) recognized by each individual increased significantly with increases in the level of malaria exposure.

**Conclusion:**

The multiplex assay developed here provides a useful tool to evaluate immune responses to multiple Ags in large populations, even when only small amounts of serum are available, or Ab titres are low, as in case of travellers. Finally, the relationship of Ab responses with malaria endemicity levels provides a way to monitor exposure in differentially exposed autochthonous individuals from various endemicity areas, as well as in travellers who are not immune, thus indirectly assessing the parasite transmission and malaria risk in the new eradication era.

## Background

Malaria is a major threat in tropical and sub-tropical regions, with nearly 50% of the world population exposed to different degrees, and an estimated 250 million people suffer annually from the disease [1]. Despite the adoption of effective interventions like artemisinin-based combination therapies, malaria is still a worldwide threat mainly due to the increasing prevalence of drug-resistant strains, the increasing risk of transmission in countries where malaria control has been reduced, and increased travel and migration [2]. Thus, malaria remains a major public health problem in the 109 endemic countries [3], as well as in other regions like Europe, where malaria due to travel is responsible for ca. 10,000 reported cases each year [4].

Diagnosis of malaria exposure and prevalence, along with the efficacy of anti-vectorial strategies and anti-malarial control measures taken by travellers, are key factors in disease control and management, though they are often neglected issues in infectious diseases related to poverty, as is malaria [5]. Some indicators that help in monitoring these factors are the incidence of clinical malaria cases and the estimation of the exposure to vector bites. However, such methods for monitoring malaria impact can be time-consuming, subjective and impractical. On the other hand, serological tools can be employed for this purpose with higher consistency and efficacy and less cost and time [6]. Indeed, seroconversion rates for malarial blood stages and pre-erythrocytic Ags correlate closely with levels of exposure to *P. falciparum *[7]. Thus, the Ab immune response against *Plasmodium *Ags can be used as one means to evaluate the exposure to malaria in travellers, even when they take anti-malarial chemoprophylaxis [8]. Furthermore, evaluation of the human response to arthropod salivary antigens could be an epidemiological indicator of exposure to vector bites, as described for the *P. falciparum *vector *A. gambiae *[9].

Standard seroepidemiological approaches include indirect immunofluorescence (IF) and ELISA tests, which are labourious and have disadvantages, such as the need for large amounts of serum and the limited number of Ags that can be included in the test at one time [10]. Currently, multiplex bead assays, such as Luminex technology [11], are preferred for high-throughput screening [12] because they are cost- and time-effective and minimize the sample volume requirements [13]. Moreover, they have been described to have similar or improved sensitivity relative to ELISA assays [13,14], and have proved useful as a tool for the detection of serum Abs directed against infectious pathogens [10,15,16].

The objectives of the present study were to set up a multi-Ag assay based on multiplex technology in order to analyse Ab responses against 13 *P. falciparum *pre-erythrocytic peptides and two *A. gambiae *salivary peptides and to examine if individuals exposed to different levels of malaria endemicity could be differentiated.

Most of the *P. falciparum *Ags included in the present study have already been considered as malaria vaccine candidates [17]. The pre-erythrocytic Ag circumsporozoite protein (CSP) is only actively expressed during the sporozoite stage and is generally used as a reference for the serological estimation of *P. falciparum *exposure [18-20]. Thrombospondin-related anonymous protein (Trap) [21] Ag is expressed on the surface of the sporozoite and is also reportedly associated with repeated malaria exposure [22]. Liver stage antigen-1 (LSA-1) [23] is expressed during the hepatic stage of *P. falciparum *infection. Ab responses against LSA-1 derived peptides have been previously shown to correlate with the cumulative exposure to malaria transmission [24]. Liver stage antigen 3 (LSA-3) [25], sporozoite threonine- and asparagine-rich protein (Starp) [23] and sporozoite- and liver-stage antigen (Salsa) are expressed at both the sporozoite and hepatic stages [26] and have been reported as antigenic [26,27]. SR11.1 Ag corresponds to a unique subregion of the megaprotein Pf 11.1 (Brahimi K., et al. unpublished data; Perlaza B.L., et al. unpublished data), and it is expressed at sporozoite and liver stages. Glutamate-rich protein (Glurp) Ag is expressed at each stage of parasite life in the human host and has been shown to be antigenic [28,29].

Studies among people living in areas endemic to malaria [30] and among travellers transiently exposed have shown that mosquito saliva is immunogenic for *A. gambiae*-specific Ags [9]. In particular, the Ab response against gSG6 (*A. gambiae's *salivary gland 6) has been described as useful for the evaluation of the intensity of human-*Anopheles *contact [31].

## Methods

### Sera samples

The study was conducted on three different populations: non-exposed, transiently exposed and regularly exposed to *A. gambiae *bites and *P. falciparum*. Twenty-one serum samples from French adults, who have never been in countries endemic to malaria and *A. gambiae*, were used as unexposed negative controls. The transiently exposed group consisted of sera from 124 French soldiers who lived in Ivory Coast between March and June 2004 [32]. The exposed group consisted of 253 people living in the Senegalese villages of Dielmo (13°45'N, 16°25'W; 82 individuals, 45 of whom were adults sampled in March 1995), Ndiop (13°14'N, 16°23'W; 86 individuals, 40 of whom were adults sampled between March and June 1995) and Diama (16°13' N, 16°23'W; 85 individuals, 38 of whom were adults). These populations were exposed to high (Dielmo, about 200 infective bites/person/year) [33], moderate (Ndiop, about 20 infective bites/person/year) [34] and low (Diama, about 2 infective bites/person/year) [35-37] malaria levels, with *A. gambiae *as one of the main vectors.

For the calculation of the seropositivity threshold, the means and standard deviations (SDs) of Ab intensity of the negative control group for all Ags were estimated. The lower limit of positivity for each Ab was taken as mean + 3.09 SD of the negative control group values. Under the hypothesis of a Normal distribution, values above this limit are expected in less than 1/1000 negative individuals.

The protocol was approved by the ethical committee of Marseille (France) and by the Senegal National Ethics Committee (Dakar, Senegal). The informed consent of each participant was obtained at the beginning of the study, after a thorough explanation of its purpose.

### Peptides

As peptides with molecular weights smaller than 3000 g/mol proved to couple poorly to beads (unpublished data), *P. falciparum *peptide Ags (Table 1) were synthesized with an added N-terminal cysteine residue and covalently coupled with the BSA (bovine serum albumin, Sigma-Aldrich, St. Louis, USA) by Genepep (Ales, France), and were stored in aliquots at -20°C. Purity of these Ags was estimated at or above 83% by HPLC and mass spectrometry.

**Table 1:** Sequences of peptides used in the study

Peptide	Sequence (N-terminal to C-terminal)	g/mol	Spectral address	Reference
**Lsa1-41**	LAKEKLQEQQSDLEQERLAKEKLQEQQSDLEQERLAKEKEKLQC	5338,84	10	[23]
**Lsa1J**	ERRAKEKLQEQQSDLEQRKADTKKC	3087,36	15	[23]
**Lsa3NR2**	VLEESQVNDDIFNSLVKSVQQEQQHNVC	3271,46	20	[25]
**Lsa3RE**	VESVAPSVEESVAPSVEESVAENVEESVC	3032,12	25	[25]
**Glurp**	EDKNEKGQHEIVEVEEILC	2282,39	30	[28]
**GlurpP3**	EPLEPFPTQIHKDYKC	1986,19	40	[29]
**Salsa1**	SAEKKDEKEASEQGEESHKKENSQESAC	3164,15	45	[26]
**Salsa2**	NGKDDVKEEKKTNEKKDDGKTDKVQEKVLEKSPKC	4060,39	50	[26]
**Trap1**	DRYIPYSPDRYIPYSPDRYIPYSPC	3138,44	55	[21,22]
**Trap2**	CHPSDGKCNCHPSDGKCNC	2046,2	60	[21,22]
**StarpR**	STDNNNTKTISTDNNNTKTIC	2340,34	65	[27]
**CSP**	NANPNANPNANPNANPNVDPNVDPC	2598,62	70	[19]
**SR11.1**	EEVVEELIEEVIPEELVLC	2254,46	75	[8]
**Saliv1**	EKVWVDRDNVYCGHLDCTRVATFC	2871,16	80	[46]
**Saliv2**	ATFKGERFCTLCDTRHFCECKETREPLC	3365,79	85	[46]

The gSG6 peptide was designed using bioinformatics to maximize its *Anopheles *specificity and antigenicity, it was then synthesized and purified (>80%) by Genosys (Sigma-Genosys, Cambridge, UK) and then BSA-conjugated (N-terminal) [31].

### Covalent coupling of Ags to beads

Carboxylated Luminex beads with different fluorescences (Biorad Inc, CA, USA) were covalently coupled with BSA only or 15 peptide-BSA complexes using a modification of the protocol from Luminex Corporation (Austin, TX, USA) as follows. Beads (5 × 10^6 ^per assay condition) were transferred into 1.5 ml tubes, vortexed, sonicated (Bandelin, Berlin, Germany) and then precipitated by centrifugation at 8,000 g for 2 min. Beads were washed with 400 μl of distilled water and pellets were resuspended in 80 μl of activation buffer (0.1 M NaH_2_PO_4_, pH 6.2). Beads were then activated using 10 μl of 1-ethyl-3-[3dimethylaminopropyl] carbodiimide hydrochloride (EDC, Pierce Biotechnology, Rockford, USA) and N-hydroxysulfosuccinimide (Sulfo-NHS, 50 mg/mL, Pierce Biotechnology), vortexed and incubated at room temperature for 20 min in the dark (IKA, Staufen, Germany). Activated beads were washed twice with 400 μl of coupling buffer (50 mM of 2-[N-morpholino] ethanesulfonic acid (MES) monohydrate pH 5, Sigma-Aldrich) and resuspended in 400 μl of coupling buffer prior to peptide addition. For optimization purposes, three different concentrations of peptides (0.075, 0.3 and 1.2 nmol) were added to the beads. Beads and peptides were vortexed, sonicated and then incubated for 2.5 h in the dark, with shaking at room temperature. Coupled beads were blocked for 30 min with shaking in the dark using 500 μl of PBS-TBN (5% BSA, 0.15% Tween-20 and 0.05% sodium azide, pH 7.4, Sigma-Aldrich) buffer. Coupled beads were washed with 1 ml of PBS-TBN buffer, resuspended in 200 μl of the same buffer and stored at 4°C in the dark. Four aliquots of 5 × 10^6 ^beads were prepared for each peptide and mixed for homogenous coupling.

### Bead-based assay

Ag-coated beads were thoroughly resuspended by vortexing and sonication, and were diluted in equal volumes of PBS and MFIA (Multiplexed Fluorescence ImmunoAssay) diluents (Charles River Laboratories Inc, MA, USA) at a final concentration of 80 beads/μl per peptide. The 1.2-μm filter-bottom 96-well microtitre plates (MSBVS 1210, Millipore, MA, USA) were pre-wetted with washing buffer (0.15% Tween 20, and 5% BSA in PBS pH 7.4) using a vacuum manifold (Millipore). Equal volumes of beads and sera (diluted from 1:50 to 1:3200 in equal volumes of PBS and MFIA diluents) were added to the wells. Plates were incubated at room temperature in the dark for 1 h with shaking at 500 rpm. After incubation, plates were washed eight times with 200 μl of washing buffer, then 100 μl of the secondary Ab (R-phycoerythrin F(ab')_2 _fragment of goat anti-human IgG, Interchim, Montluçon, France) diluted 1:500 (1 μg/ml), was added to each well. After 30 min of incubation in the dark at room temperature with shaking, plates were washed as described previously. Beads were resuspended in 100 μl of a solution of 5% BSA-PBS, pH 7.4 and finally analysed on Luminex system. The system was set to read a minimum of 100 beads per spectral address, and results were expressed as the median fluorescent intensity (MFI).

### ELISA assay

A random sub-sample of 30 sera representative of those used for the bead-based assays (*i.e. *from the four groups : 8 travellers, 8 adults from Diama, 5 adults from Ndiop and 9 adults from Dielmo) were also tested against the same antigens by conventional ELISA using a method previously described [8] that has been slightly modified, *i.e. *using peptide-BSA complexes that were linked to the beads and BSA-coated wells as control wells, i.e. without peptide.

### Statistical analysis

Statistical analyses were performed with the R software package version 2.8 [38]. Proportions of Ab-positive individuals, Ab levels and number of Ags recognized by each person were analysed using the chi-squared test, Fisher's exact test, Kruskal-Wallis test and Spearman's rank correlation test, where appropriate. Differences were considered statistically significant when p-values were less than 0.05.

## Results

### Optimal serum dilution and Ag concentration

**Figure 1:**
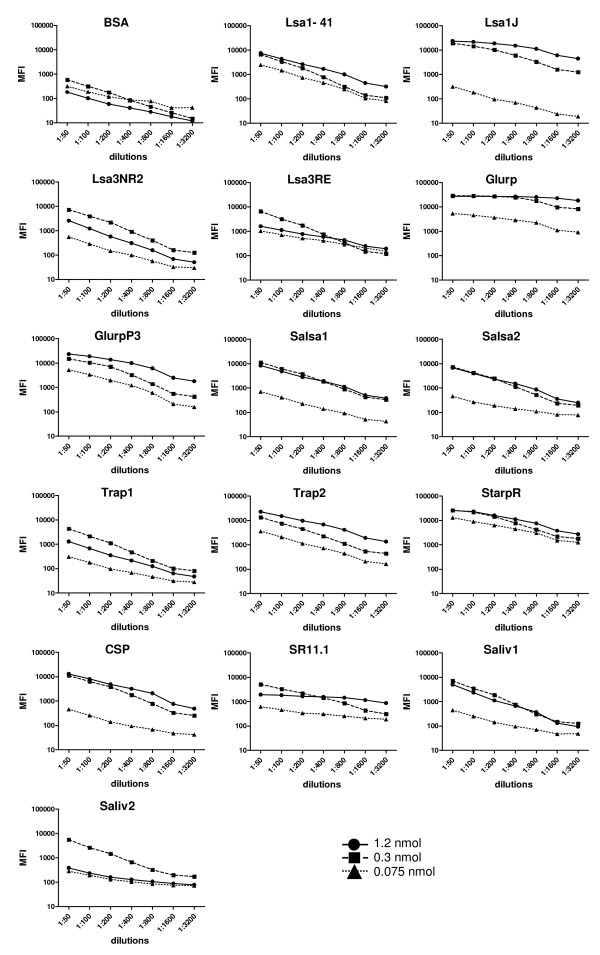
**Determining the optimal Ag concentration to be used for bead coating and the optimal serum dilution**. The figure shows MFI values obtained from the serum of an exposed adult from Dielmo village at seven different dilutions (from 1:50 to 1:3200) with beads coated with three concentrations (0.075, 0.3 and 1.2 nmol) of synthetic peptides. All peptides were detected by the tested serum and their signals were much higher than the background signal against BSA-coated beads (upper left graph).

**Figure 2:**
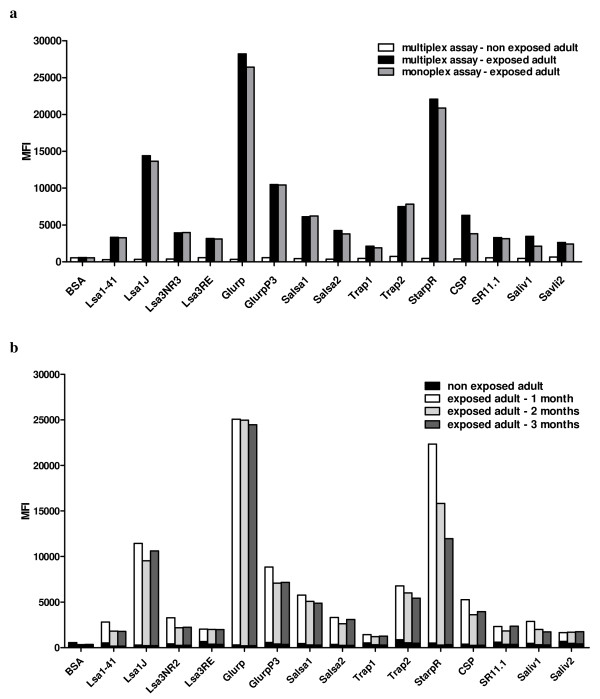
**Comparison between exposed and unexposed adults: monoplex and multiplex assays showing coated bead-stability over time**. In the upper panel (a), the same serum from the exposed individual from Dielmo village (black bars) tested in figure 1 and serum from an unexposed adult (white bars) were compared at a dilution of 1:100 by the multiplex assay. The assay allows a clear separation of peptide-specific MFI between the two samples, whereas the backgrounds (MFI values with BSA-coated beads) were equivalent. The serum from the exposed individual from Dielmo village was also tested for the same Ab responses by the monoplex assay with single peptide-coated beads (gray bars). Results with monoplex and multiplex assays were almost identical (correlation coefficient R^2 ^= 0.9896). The lower panel (b) shows results obtained using peptide-coupled beads after 1 (white bars), 2 (light gray bars) and 3 (dark gray bars) months of storage at 4°C against the 1:100 diluted serum from the exposed adult from Dielmo village and the 1:100 diluted serum from the unexposed adult (black bars, superimposed to the corresponding time delay). MFIs were consistent for all Ags, except StarpR (the correlation coefficient comparing values using coated beads after 1 and 3 months for each peptide, except StarpR, was R^2 ^= 0.9874).

To determine the optimal Ag amount for bead coating, three concentrations (0.075, 0.3 and 1.2 nmol) of synthetic peptides were coupled to 5 × 10^6 ^beads. BSA-coated beads were included as background control. Coated beads were then incubated with the serum from an exposed adult from Dielmo village (Figure 1). To determine the optimal serum dilution for the analysis, seven dilutions (from 1:50 to 1:3200) of the serum from the exposed individual from Dielmo village were tested in parallel (Figure 1) together with the serum from an unexposed adult (white bars Figure 2a for 1:100 dilution).

As shown by the distribution of Ab MFI in figure 1, all peptide concentrations were detected by the tested serum. For the majority of peptides, the signal saturated when 0.3 nmol of Ag was used. The lowest (0.075 nmol) and the highest (1.2 nmol) Ag concentrations did not yielded optimal results, the latter probably due to Ab competition. Background values (MFI values using BSA-coated beads) were low, compared to MFI values obtained with other peptides, reaching a maximum of 600.

With regard to serum dilutions, the highest MFI values were observed with the lowest 1:50 dilution (Figure 1). Nevertheless a working dilution of 1:100 was preferred as being less sera consuming, and because as shown in Figure 2a, 1:100 sera dilutions led to a clear separation of MFI between the exposed (black bars, for multiplex assay) and non exposed (white bars) individuals. MFI values obtained with the serum from the exposed individual from Dielmo village ranged from 2130 (Trap1) to 28236 (Glurp), whereas with the serum from the unexposed individual reached a maximum of 719 (Trap2). As expected, the background (MFI values with BSA-coated beads) was equivalent for both sera.

### Comparison between monoplex and multiplex assays

The Ab responses detected by multiplex assay (all Ags tested in the same assay) were compared to those detected by monoplex assay (each Ag tested in a separate assay). As shown in Figure 2a, the results obtained from the exposed individual's sera (1:100 dilution) from Dielmo village incubated with single Ag-coated beads (gray bars) or with equal amounts of the 15 tested Ag-coated beads (black bars) were essentially identical. MFI values, obtained by monoplex and multiplex assays shown in figure 2a, were compared using a linear regression curve, and a correlation coefficient of R^2 ^= 0.9896 was determined. The high level of correlation between the two assays indicated that individual peptides coupled to beads did not compete for the Abs in the serum. Similar results were obtained regardless of serum dilution, with correlation coefficients for all peptides as follows: dilution 1:50, R^2 ^= 0.995; 1:200, R^2 ^= 0.997; 1:400, R^2 ^= 0.999; 1:800, R^2 ^= 0.998; 1:1600, R^2 ^= 0.998; and 1:3200, R^2 ^= 0.999.

### Coating stability over time

The stability of Ag-coupled beads after 1, 2 and 3 months storage at 4°C was examined by testing against sera from the exposed and unexposed adults; results are shown in Figures 1 and 2a. As reported in Figure 2b, the results were consistent for all Ags, indicating that the storage time had no effect on coupling stability, except for the StarpR peptide. The correlation between the results obtained with beads stored for 1 and 3 months was high, and the correlation coefficient for all peptides except StarpR was R^2 ^= 0.994. Therefore, StarpR-coupled beads had to be used within 1 month after preparation, whereas all other Ags-coupled beads can be stored for longer periods before use.

### Proportion of seropositive individuals according to malaria exposition level

**Figure 3:**
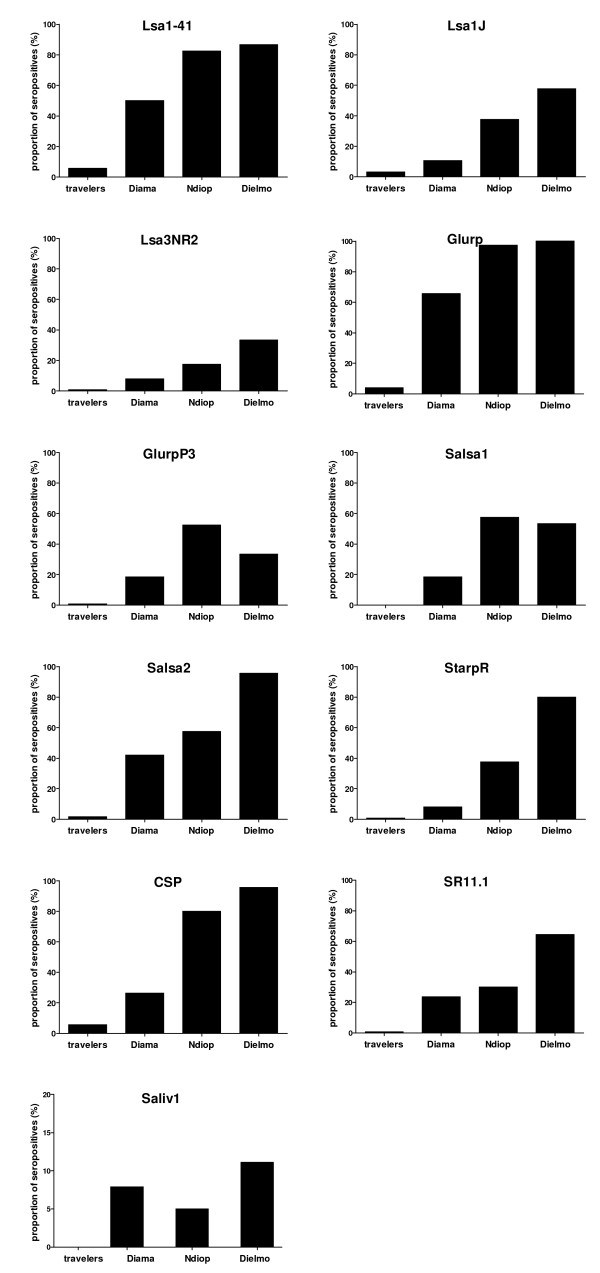
**The proportion of seropositive individuals increases with malaria exposure level**. Among the adults (124 travelers, 45 from Dielmo, 40 from Ndiop and 38 from Diama), the proportion of seropositives for Lsa1-41 (p < 0.001), Lsa1J (p < 0.001), Lsa3NR2 (p < 0.001), Glurp (p < 0.001), GlurpP3 (p < 0.001), Salsa1 (p < 0.001), Salsa2 (p < 0.001), StarpR (p < 0.001), CSP (p < 0.001), SR11.1 (p < 0.001) and Saliv1 (p = 0.001) peptides differed significantly between groups (Fischer's exact test). In travellers, no Abs against Saliv1 Ag were detected; Abs were detected in exposed adults (three far left bars). 95% confidence intervals are shown in table 2.

In total, 377 individuals exposed to malaria from areas with different endemicities were tested for all of the studied Ags. Among adults, for the Ags Lsa1-41, Lsa1J, Lsa3NR2, Glurp, Salsa2, StarpR, CSP and SR11.1, the proportion of seropositive adults increased significantly with the malaria endemicity (Figure 3, bars from left to right p ≤ 0.01). For GlurpP3 and Salsa1 Ags the increase was observed with the exception of the higher exposed group from Dielmo (Figure 3, comparison among the three far left bars p ≤ 0.01). For the *A. gambiae *Saliv1 Ag (Figure 3, bottom left), the proportion of seropositives in transiently exposed adults was undetectable, while Abs were detected in exposed adults living in villages endemic for malaria (no significant difference among individuals from Ndiop, Diama and Dielmo). For the four remaining Ags (Lsa3RE, Trap1, Trap2, Saliv2), the proportion of seropositives in the groups was too low to yield any significant difference. All proportions of seropositive adults for the 15 tested Ags are listed in Table 2, with the respective CI 95% value.

**Table 2:** Number and proportion of seropositives adults (%)

	Travelers (N = 124)				Diama (N = 38)				Ndiop (N = 40)				Dielmo (N = 45)			
	pos	%	95%CI		pos	%	95%CI		pos	%	95%CI		pos	%	95%CI	
LSA1-41	7	5,6%	2,3%	11,3%	19	50%	33%	67%	33	83%	67%	93%	39	87%	73%	95%
LSA1-J	4	3,2%	0,9%	8,1%	4	11%	3%	25%	15	38%	23%	54%	26	58%	42%	72%
LSA3-NR2	1	0,8%	0,0%	4,4%	3	8%	2%	21%	7	18%	7%	33%	15	33%	20%	49%
LSA3-RE	0	0,0%	0,0%	2,9%	0	0%	0%	9%	0	0%	0%	9%	3	7%	1%	18%
GLURP	5	4,0%	1,3%	9,2%	25	66%	49%	80%	39	98%	87%	100%	45	100%	92%	100%
GLURP P3	1	0,8%	0,0%	4,4%	7	18%	8%	34%	21	53%	36%	68%	15	33%	20%	49%
SALSA1	0	0,0%	0,0%	2,9%	7	18%	8%	34%	23	58%	41%	73%	24	53%	38%	68%
SALSA2	2	1,6%	0,2%	5,7%	16	42%	26%	59%	23	58%	41%	73%	43	96%	85%	99%
TRAP1	0	0,0%	0,0%	2,9%	0	0%	0%	9%	3	8%	2%	20%	2	4%	1%	15%
TRAP2	0	0,0%	0,0%	2,9%	0	0%	0%	9%	0	0%	0%	9%	1	2%	0%	12%
STARP-R	1	0,8%	0,0%	4,4%	3	8%	2%	21%	15	38%	23%	54%	36	80%	65%	90%
CSP	7	5,6%	2,3%	11,3%	10	26%	13%	43%	32	80%	64%	91%	43	96%	85%	99%
SR11.1	1	0,8%	0,0%	4,4%	9	24%	11%	40%	12	30%	17%	47%	29	64%	49%	78%
SALIV1	0	0,0%	0,0%	2,9%	3	8%	2%	21%	2	5%	1%	17%	5	11%	4%	24%
SALIV2	1	0,8%	0,0%	4,4%	0	0%	0%	9%	0	0%	0%	9%	0	0%	0%	8%

pos. : seropositve. 95%CI : 95% confidence interval.

The proportion of seropositive children (<10 years old) and young people (from 10 to 20 years old) within the three groups living in endemic areas was also compared. The comparison showed that the proportion increased significantly with age and according to the level of malaria exposure for the following Ags: Lsa1-41, Lsa1J, Lsa3NR2, Glurp, GlurpP3, Salsa1, Salsa2, StarpR, CSP, SR11.1 and Saliv1 (p ≤ 0.001).

### The Intensity of specific Ab response also increases as a function of malaria exposure

**Figure 4:**
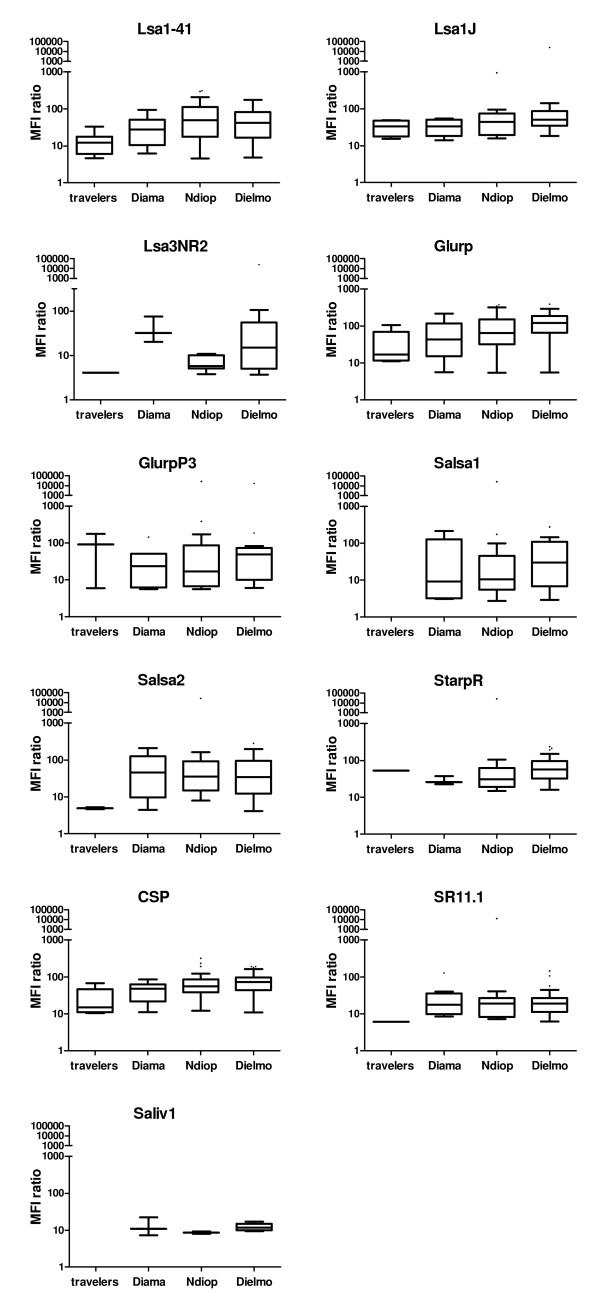
**MFI response in seropositive individuals increased with exposure to malaria**. The distribution of corrected MFI (median fluorescent intensity) values (MFI_Ag_/MFI_BSA_) within groups is shown for the peptides Lsa1-41, Lsa1J, Lsa3NR2, Glurp, GlurpP3, Salsa1, Salsa2, StarpR, CSP, SR11.1 and Saliv1. The difference between groups was significant for Lsa1-41 (p = 0.02), Glurp (p = 0.0014) and CSP (p = 0.002), Kruskal-Wallis test. The number of seropositive individuals per group is presented in table 2.

The mean of MFI values of seropositive adults are presented in Figure 4. The results shown have been corrected for the MFI for each Ag (MFI_Ag_/MFI_BSA_). The median specific Ab intensity increased significantly with the malaria exposure level for Lsa1-41 (p < 0.02), Glurp (p < 0.002), and CSP (p = 0.002) Ags. Moreover, a similar trend was observed for some of the remaining Ags, as shown in Figure 4. Comparisons for Lsa3RE, Trap1, Trap2, and Saliv2 were not significant.

### Number of Ags that showed seropositivity

**Figure 5:**
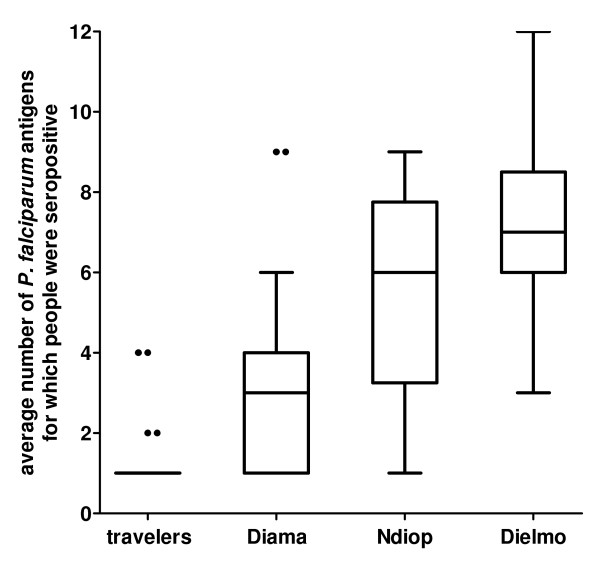
**The number of *P. falciparum *peptides that produced seropositive results increased with malaria exposure**. The distribution of the total number of *P. falciparum *peptides for which each individual was seropositive is shown. The number of recognized peptides increased significantly with the malaria exposure level (p < 0.0001). The middle lines of the boxes represent the median distribution; the upper lines indicate the upper quartiles and the lower lines show the lower quartile. The upper and the lower lines outside the boxes represent the upper and lower non-outsider observations, respectively, while the dots are the outliers.

The number of Ags in the different groups that produced seropositive results was investigated. Figure 5 shows the distribution of the mean number of *P. falciparum *Ags for which adults within the groups were seropositive. The higher the exposure level to malaria, the higher the mean number of *P. falciparum *Ags that produced seropositivity. In the travellers group, the median number of *P. falciparum *Ags for which individuals were seropositive was one, whereas it was three for individuals from Diama, six for individuals from Ndiop and seven for individuals from Dielmo. The difference between groups was significant (p < 0.0001).

### Correlation of the evaluations of Ab responses by bead-based assays and ELISA

There were significant correlations of the evaluations of the Ab responses obtained by bead-based assay and conventional ELISA assay with the peptides Lsa1-41, Lsa1-J, Lsa3-NR2, Glurp, Glurp P3, Salsa1, Salsa2, Trap1, Starp-R, CSP and Saliv1 (Spearman's rank correlation coefficient ranging from 0.79 to 0.35, mean coef. 0.58; 0.0001 ≤ p-values < 0.05; additional file 1). The correlations were not significant for the Lsa3-RE, Trap2, SR11.1 and Saliv2 peptides, possibly in relation with a lack of antigenicity of the peptites coated on beads or with the narrowness of the differences in Ab responses to these antigens between the sera used for the present analysis.

## Discussion

In this study, a multiplex assay to simultaneously measure responses to 13 peptides derived from pre-erythrocytic *P. falciparum *Ags and 2 peptides derived from one *A. gambiae *salivary Ag has been developed.

Though multiplex immunoassays have already proven to be useful in serological [39-42] and malaria research [10,43], the present multiplex assay is the first to include 15 peptides simultaneously and to combine *P. falciparum *and *A. gambiae *Ags.

No interference between bead sets was observed in the present study (Figure 2a) or in work from others [10]. Furthermore, previous reports have shown that multiplex results are consistent with those obtained by ELISAs and that both methodologies are equally sensitive [14,44]. The multiplex assay is a flexible technique, allowing new targets to be included when required. The simultaneous evaluation of multiple Abs leads to a reduction in both the time needed for the measurement and in its cost. The small volume of serum required is an important criterion, as it is fitted to the screening against several Ags of large populations of children, from whom minimal amounts of blood can be obtained. Therefore, multiplex assays can reliably test Abs to multiple Ags at one time in a fast and affordable manner. The high price of the Luminex machine prevents however that it is as widespread as the ELISA machines.

Thirteen pre-erythrocytic peptides from *P. falciparum *were included in the assay; such peptides are useful for monitoring the exposure to the malaria parasite. Using peptides in place of intact proteins in such immunological essay could lead to a loss of sensitivity (*i.e. *a restriction on the number of epitopes) and may on occasion give unexpected cross-reactivities. However, the peptides chosen for the present study had been previously compared to recombinant proteins in ELISA essays and were shown to give consistent results. A high prevalence of Ab response to pre-erythrocytic Ags has been found in individuals living in malaria endemic areas [23,26,27], and the level of exposure to *P. falciparum *correlates with prevalence rates for those Ags [7]. In agreement with previous data, the present results showed that for some of these peptides, such as Lsa1-41, Lsa1J, Lsa3NR2, Glurp, Salsa2, StarpR, CSP and SR11.1, the proportion of seropositive individuals clearly increases with malaria exposure levels (Figure 3), and higher Ab response rates were seen with the Lsa1-41, Glurp, Salsa2, and CSP peptides. This data suggests that such Ags can be considered as possible biomarkers of exposure, and further studies in such direction would be valuable. Moreover, for all peptides shown in Figure 3, the proportion was higher in exposed individuals living in malaria endemic countries, compared to transiently exposed travellers. This trend was similar for median fluorescence intensities of Lsa1-41, Glurp, Salsa1, Salsa2 and CSP SR11.1 peptides (Figure 4). The very low responses recorded towards LSA3-RE are in sharp contrast with the high prevalence rates and titers obtained previously when using the same peptide in ELISA assays ([22], and unpublished observation in the same study populations investigated here). This may reflect alterations of antigenicity related to the coating to BSA or to multiplex beads, and raise doubts about the results obtained with that particular peptide in the multiplex assay.

When evaluating the distribution of the mean number of peptides against which individuals were seropositive, the value was found related to the level of malaria endemicity to which they were exposed. Figure 5 shows that such measurements can clearly stratify individuals based on their exposure levels, with a clearly higher number of peptides recognized by individuals exposed to high malaria transmission.

In addition to pre-erythrocytic *P. falciparum *Ags, the assay also included two peptides derived from one *A. gambiae *salivary Ag. As previously shown, the evaluation of immune responses to mosquito salivary Ags can indicate exposure to vector bites. Studies have demonstrated that children living in malaria endemic regions develop Abs against the vector's salivary proteins [30]. Thus, Abs against *A. gambiae *salivary Ags represent an immunological marker of exposure to malaria vector [30]. One of these Ag, the gSG6 salivary protein, was first described in 1999 [45]. The gSG6 Ag is highly conserved among *Anopheles *species. It was reported to be potentially antigenic in travellers briefly exposed to *Anopheles *bites and was more recently confirmed as antigenic in Senegalese children [31].

The two peptides derived from gSG6 protein included in the assay (Saliv1 and Saliv2) were antigenic, but the intensity of their IgG responses was peptide-dependent, where Saliv1 showed the higher response [46]. In the present study, Saliv1 was the target of an Ab response only in regularly exposed individuals (Figures 3 and 4, and Table 2). The results of the present study indicated that the immune response to Saliv1 peptide could be used to differentiate individuals, based on their exposure to *Anopheles *bites, short-term exposure in travellers and long-term exposure in people living in endemic countries. Furthermore, immune responses against such peptides are difficult to detect in briefly exposed travellers, though a response was observed when the Abs were tested directly against mosquito saliva [9].

It has been reported recently that the gSG6-P1 peptide (corresponding to Saliv1) can be used to evaluate low-level exposures to *Anopheles *bites, notably in Senegalese children ≥2 years of age [31]. Unfortunately, in the present study population, the number of young children was too low to confirm these findings. Furthermore, the low-level exposure group (travellers) was exposed to *Anopheles *bites at a lower rate compared to Senegalese children due shorter exposure times and systematic use of anti-vectorial devices (impregnated bed nets, long-sleeved battledresses and repellents). This factor, together with the different assay used - multiplex at a 1:100 dilution of sera in our case and ELISA at a 1:20 dilution of sera in the study performed by Poinsignon *et al *[31] - could explain why Abs against Saliv1 were not detected in the travellers group. Further, the epitopes used could be less representative of the native ones present in humans and, therefore, less antigenic.

## Conclusions

The multiplex technology developed in the present study is a reliable and fast method to test immune responses to *P. falciparum *and *A. gambiae *salivary Ags in large populations, allowing efficient identification of putative protective Abs. Furthermore, the association of Ab responses to malaria exposure levels provides a way to monitor exposure in differentially exposed indigenous people, as well as in travellers not immune to the parasite, allowing the assessment of *P. falciparum *transmission and malaria risk.

## Competing interests

The authors declare that they have no competing interests.

## Authors' contributions

EA carried out statistical analysis and interpretation of the data, drafted and revised the manuscript. CD developed the techniques, carried out all the immunological investigations, carried out initial statistical analysis and revised the manuscript. AP carried out the ELISA analysis. ATB, AT, CS, J-FT, EO-P, FR and JBS collected data and sera. TF, FR, AP, EO-P and PD contributed to the design of the method and to the choice of the antigens and peptides. KP produced the peptides. CR was the principal investigator of the study, conceived the research programme, participated in the development of the technique, statistical analysis, interpretation of the data and revision of the manuscript. All authors read and approved the final manuscript

## Supplementary Material

Additional file 1 **Correlation between Luminex and ELISA results obtained with 30 sera**. The Spearman correlation coefficient and the Pearson correlation coefficient between Luminex and ELISA results have been estimated on 30 sera. Click here for file
