# Supplementary material for: A multiplex assay for the simultaneous detection of antibodies against 15 Plasmodium falciparum and Anopheles gambiae saliva antigens
Source: Malar J. 2010 Nov 8;9:317. doi: 10.1186/1475-2875-9-317 (PMC2992071; doi:10.1186/1475-2875-9-317)
Supplement: Additional file 1 — Correlation between Luminex and ELISA results obtained with 30 sera. The Spearman correlation coefficient and the Pearson correlation coefficient between Luminex and ELISA results have been estimated on 30 sera. [file 1475-2875-9-317-S1.DOC]

**Additional file1:**

Correlation coefficients of Spearman and Pearson between Luminex and ELISA results obtained with 30 sera.

|  | **Spearman** |  | **Pearson** |  |
| --- | --- | --- | --- | --- |
|  | **Rho** | p-value | **R** | p-value |
| LSA1.41 | 0,79 | <0.0001 | 0,82 | <0.0001 |
| LSA1 J | 0,59 | 0,0007 | 0,67 | <0.0001 |
| LSA3 NR2 | 0,45 | 0,0133 | 0,58 | 0,0007 |
| LSA3 RE | 0,20 | 0,2904 | 0,15 | 0,4180 |
| GLURP | 0,67 | <0.0001 | 0,71 | <0.0001 |
| GLURP P3 | 0,75 | <0.0001 | 0,68 | <0.0001 |
| SALSA 1 | 0,67 | <0.0001 | 0,52 | 0,0034 |
| SALSA 2 | 0,71 | <0.0001 | 0,68 | <0.0001 |
| TRAP 1 | 0,38 | 0,0407 | 0,32 | 0,0855 |
| TRAP 2 | 0,07 | 0,7059 | 0,02 | 0,9246 |
| STARP | 0,35 | 0,0583 | 0,49 | 0,0063 |
| CSP | 0,68 | <0.0001 | 0,74 | <0.0001 |
| SR11.1 | 0,26 | 0,1600 | 0,11 | 0,5652 |
| SALIV 1 | 0,40 | 0,0277 | 0,33 | 0,0737 |
| SALIV 2 | 0,08 | 0,6903 | 0,13 | 0,5069 |
